# *Rhinacanthus nasutus* Ameliorates Cytosolic and Mitochondrial Enzyme Levels in Streptozotocin-Induced Diabetic Rats

**DOI:** 10.1155/2013/486047

**Published:** 2013-04-09

**Authors:** Pasupuleti Visweswara Rao, K. Madhavi, M. Dhananjaya Naidu, Siew Hua Gan

**Affiliations:** ^1^Department of Biotechnology, Sri Venkateswara University, Tirupati 517502, Andhra Pradesh, India; ^2^Human Genome Centre, School of Medical Sciences, Universiti Sains Malaysia, 16150 Kubang Kerian, Malaysia; ^3^Department of Biochemistry, Sri Venkateswara Medical College, Tirupati 517502, Andhra Pradesh, India; ^4^Department of Zoology, Yogi Vemana University, Kadapa 516003, Andhra Pradesh, India

## Abstract

The present study was conducted to evaluate the therapeutic efficacy of *Rhinacanthus nasutus* (*R. nasutus*) on mitochondrial and cytosolic enzymes in streptozotocin-induced diabetic rats. The rats were divided into five groups with 6 rats in each group. The methanolic extract of *R. nasutus* was orally administered at a dose of 200 mg/kg/day, and glibenclamide was administered at a dose of 50 mg/kg/day. All animals were treated for 30 days and were sacrificed. The activities of both intra- and extramitochondrial enzymes including glucose-6-phosphate dehydrogenase (G6PDH), succinate dehydrogenase (SDH), glutamate dehydrogenase (GDH), and lactate dehydrogenase (LDH) were measured in the livers of the animals. The levels of G6PDH, SDH, and GDH were significantly reduced in the diabetic rats but were significantly increased after 30 days of *R. nasutus* treatment. The increased LDH level in diabetic rats exhibited a significant reduction after treatment with *R. nasutus*. These results indicate that the administration of *R. nasutus* altered the activities of oxidative enzymes in a positive manner, indicating that *R. nasutus* improves mitochondrial energy production. Our data suggest that *R. nasutus* should be further explored for its role in the treatment of diabetes mellitus.

## 1. Introduction 

Type 1 diabetes mellitus (T1DM) is caused by the destruction of insulin-producing *β* cells in the pancreas and is usually the result of an autoimmune disease. T1DM leads to uncontrolled blood glucose levels, which are associated with long-term damage, including retinopathy, neuropathy, nephropathy, and damage to the heart and blood vessels and with multiple organ failures [1]. To date, various types of herbs and other plant materials from all over the world have been used for the treatment of diabetes mellitus. Medicinal plants are a good source of compounds with hypoglycemic effects and are important for the development of new drugs and as adjuncts to existing therapies [2]. Some of the medicinal plants such as *Panax ginseng *and* Piper longum* possess significant antihyperglycemic and antihyperlipidemic actions which have been reported to have the potential to alleviate impaired oxidative stress in diabetic rats [3, 4]. Generally, herbal products are gaining popularity due to their natural origins, the lower incidence of side effects, and their relatively lower costs relative to synthetic drugs [5], although their efficacies may still be inferior when compared to some current available treatments such as insulin and meformin.

*Rhinacanthus nasutus* (Linn) is a flowering plant that belongs to the *Acanthaceae *family and is well recognized for its remedial uses. This plant is commonly known as *Nagamalli* in Telugu, *Doddapatika* in Kannada, *Kaligai *and *Anichi* in Tamil, *Yuthikaparni* in Sanskrit, *Jupani* in Hindi, and *Gajakarni* in Marathi [6]. *R. nasutus* is also commonly known as Snake Jasmine due to the shape of its flowers. In addition, the root of this plant has been reported to be used in traditional medicine to counter the effects of snake venom [7]. Different parts of this plant have also been traditionally used for the treatment of various diseases such as diabetes, eczema, pulmonary tuberculosis, herpes, hypertension, hepatitis, and several types of skin diseases. In Thailand, *R. nasutus* has been traditionally used for the treatment of various cancers such as colon [8], cervical, and liver cancers [9].

Previously, we reported that *R. nasutus* possesses antimicrobial properties and can kill a variety of infecting organisms, in addition to exhibiting antidiabetic effects, hypolipidemic activity [10], and significant *in vitro *and* in vivo* antioxidant activities [11]. In this study, we hypothesized that *R. nasutus* may exert antidiabetic effects by ameliorating cytosolic and mitochondrial enzymes levels.

## 2. Materials and Methods

### 2.1. Collection of Plant Material

The fresh leaves of *R. nasutus* were collected from Tirumala Hills, Tirupati, and Chittoor district of Andhra Pradesh, in the period of July–October 2009. Seshachala and Tirumala Hills (Rayalaseema region, Andhra Pradesh, India), areas that are geographically located in the South Eastern Ghats, are recognized for their rich flora and fauna [12]. The plant specimen was verified to be of the correct species by Dr. Madhava Setty, a botanist from the Department of Botany, S. V. University, Tirupati. 

### 2.2. Preparation of the Extract

Fresh leaves of *R. nasutus* (500 g) were shade-dried and milled into fine powder using a mechanical grinder (TTK Prestige, Chennai, India). The powdered plant material was macerated and shaken in methanol using a bath shaker (Thermo Scientific, Mumbai, India) for 48 h. The extract was then filtered with filter paper (Whatman No. 1) and evaporated to dryness under vacuum and reduced pressure using a rotary evaporator at 40°C. The concentrate was then placed on aluminum foil before freeze drying. The residual extract was dissolved in sterile water (1 mL) before use.

### 2.3. Chemicals

Streptozotocin (STZ) was purchased from Sigma (USA). All other chemicals and reagents used in this study were of analytical grade. Glibenclamide (Sugatrol, Hyderabad, India) was purchased from a local drug store.

### 2.4. Experimental Design

Adult male Wistar rats weighing between 150 and 180 g were obtained from Sri Venkateswara Enterprises, Bangalore. They were individually housed in clean, sterile polypropylene cages under standard conditions (12 h light/dark cycles) with free access to standard chow (Hindustan Lever Ltd., Bangalore, India) and water *ad libitum*. For one week prior to the start of the experiments, the animals were acclimatized in the laboratory. The animal experiments were designed and performed in accordance with the ethical norms approved by the local Ministry of Social Justices and Empowerment, Government of India, and the Institutional Animal Ethics Committee Guidelines (Resolution no. 05/(i)/a/CPCSEA/IAEC/SVU/MDN-PVR/dt.13.09.2010).

### 2.5. Induction of Experimental Diabetes

The rats were divided into five groups of six animals each:

 Group I. Normal rats (controls: animals receiving only buffer).
 Group II. *R. nasutus*-treated normal rats (200 mg/kg/day).
 Group III. Diabetic rats (untreated).
 Group IV. *R. nasutus*-treated diabetic rats (200 mg/kg/day).
 Group V. Glibenclamide-treated diabetic rats (50 mg/kg/day).

The dose of 200 mg/kg was selected based on our previous study [13] where we found that 200 mg/kg dose gave similar effects to 250 mg/kg dose. 

The toxicity of the extract has been tested. There were no toxic effects observed. There are some previous studies where it has been reported that the plant extract has no toxic effect when used in animals at higher doses also 500 mg/kg dose [8].

Diabetes was induced by a single intraperitoneal injection of a freshly prepared STZ solution (Sigma, no. 242-646-8) (50 mg/kg in citrate buffer 0.01 M, pH 4.5) to overnight-fasted rats. Diabetes was confirmed by the presence of polydipsia and polyurea and by measuring the nonfasting plasma glucose levels 48 h after injection of STZ. Only animals that were confirmed to have blood glucose levels of greater than 250 mg/dL [1] were included. All of the animals were allowed free access to tap water and pellet show per the guidelines of the Institute Animal Ethics committee.

### 2.6. Phytochemical Analysis

Qualitative tests were conducted on the crude extract using various solvents such as hexane, ethyl acetate, methanol, and water for the different phytochemical constituents present in the plant extract based on the previous method described by Harborne [14]. 

### 2.7. Test for Glycosides (Keller-Kiliani Test)

The test for glycosides was conducted based on the method published by Kokate et al. [15]. Briefly, the extracts were dissolved in glacial acetic acid, and two drops of ferric chloride solution (5%, w/v in 90% alcohol) were added. The mixture was then transferred to a test tube containing 2 mL of concentrated sulfuric acid. The presence of a reddish brown ring between the two layers confirmed the presence of glycosides.

### 2.8. Test for Flavonoids

The test for flavonoids (lead acetate test) was conducted based on the method published by Peach and Tracey [16]. Briefly, several drops of lead acetate solution (10%) were added to the alcoholic solution of the extract and a yellow precipitate was formed.

### 2.9. Test for Phenols

The presence of phenols (ferric chloride test) was confirmed using the method published by Trease and Evans [17]. Briefly, several drops of neutral ferric chloride solution (5%, w/v in 90% alcohol) were added to the extract. A blackish green color indicated the presence of a phenolic group, indicating that the extract contained phenolic substances, which are antioxidants.

### 2.10. Biochemical Measurements

All animals were sacrificed by cervical dislocation at the end of the experiment on day 30. The liver tissues were excised at 4°C. The tissues were washed with ice-cold saline and were immediately immersed in liquid nitrogen and stored at −80°C for further biochemical analysis. The activities of selected cytosolic enzymes were then assayed. 

The activity of lactate dehydrogenase (LDH) was measured using the method adapted from Prameelamma and Swami [18] with slight modifications. The activity of glucose-6-phosphate dehydrogenase (G6PDH) was measured using the method established by Lohr and Waller [19]. The levels of mitochondrial enzymes including succinate dehydrogenase (SDH) were assayed using a modified method published by Nachlas et al. [20]. The activity of glutamate dehydrogenase GDH) was determined by the method established by Lee and Lardy [21]. All enzymatic assays in this study were performed using crude liver homogenate.

### 2.11. Statistical Analysis

The results were expressed as the mean ± SD (*n* = 6). Statistical analysis was performed using one-way analysis of variance (ANOVA) followed by Tukey's test. *P* < 0.05 was considered statistically significant.

## 3. Results 

### 3.1. Phytochemical Analysis of Different Extracts of *R. Nasutus*

*R. nasutus* extracts produced using different solvents were screened for their phytochemical contents. Several compounds were confirmed to be present in the various types of extract (Table 1). Steroids were present in all of the extracts, whereas triterpenes and saponins were present only in the hexane and ethyl acetate extracts. Flavonoids, tannins, and carbohydrates were present in the aqueous extract. Glycosides were not detected in any of the extracts. Because the methanolic extract contained the highest concentrations of compounds, it was selected for further analysis.

### 3.2. The Effects of *R. nasutus* Extract on Cytosolic and Mitochondrial Enzymes

**Figure 1:**
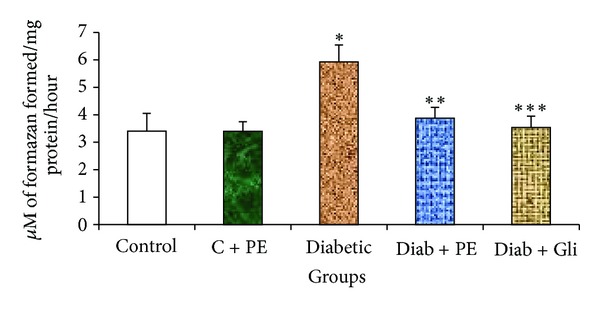
Changes in LDH activity in the liver tissue of experimental rats. Bars with the same superscripts do not differ significantly at *P* < 0.05. C = control, PE = plant extract, Diab = diabetic, Gli = glibenclamide.

The oral administration of the *R. nasutus* extract to diabetic rats significantly reduced the LDH activity relative to that of diabetic control rats. This reduction was also observed for diabetic rats treated with glibenclamide, indicating that the activity of *R. nasutus* is similar to that of glibenclamide. However, no difference in LDH activity was observed in normal rats treated with *R. nasutus* (Figure 1), indicating that the effects of *R. nasutus* reverse the changes in LDH activity only in diabetic rats.

**Figure 2:**
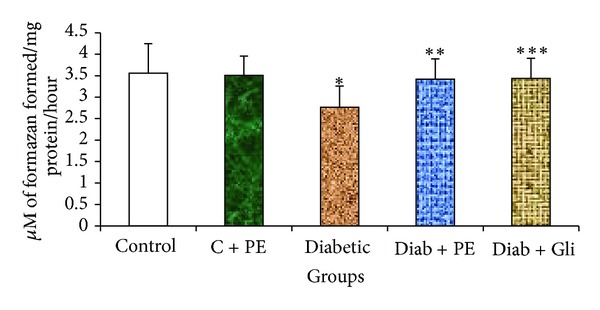
Changes in the SDH level in the liver tissue of experimental rats. Bars with the same superscripts do not differ significantly at *P* < 0.05. C = control, PE = plant extract, Diab = diabetic, Gli = glibenclamide.

The activity of the mitochondrial marker enzyme SDH was significantly decreased in the diabetic rats. In this study, we demonstrated that the decrease in SDH activity among the diabetic rats was ameliorated by treatment with the *R. nasutus* extract. The increase in SDH activity in *R. nasutus* extract-treated diabetic rats was similar to the augmentation of the SDH activity by glibenclamide (Figure 2), indicating that *R. nasutus* has protective effects on SDH activity similar to those of glibenclamide. 

**Figure 3:**
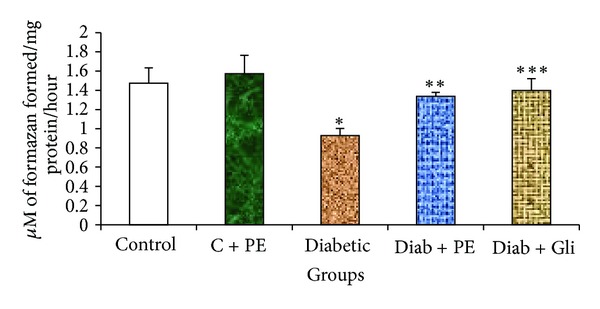
Changes in the GDH level in the liver tissue of experimental rats. Bars with the same superscripts do not differ significantly at *P* < 0.05. C = control, PE = plant extract, Diab = diabetic, Gli = glibenclamide.

STZ injection resulted in a significant decrease in GDH activity among animals in the diabetic group, a change that was not observed in normal control rats. Interestingly, we found a higher level of GDH activity in *R. nasutus-*treated diabetic rats compared with diabetic control rats. Treatment with the *R. nasutus* extract resulted in an improvement in the GDH activity that was equal to the improvement observed in the glibenclamide-treated diabetic rats (Figure 3), again indicating that the extract has protective effects on GDH activity similar to those of glibenclamide.

**Figure 4:**
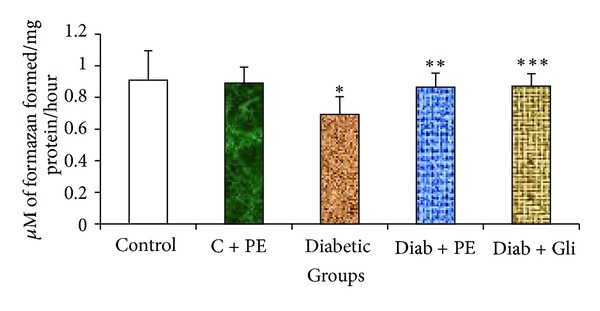
Changes in G6PDH in the liver tissue of experimental rats. Bars with the same superscripts do not differ significantly at *P* < 0.05. C = control, PE = plant extract, Diab = diabetic, Gli = glibenclamide.

The G6PDH activity in diabetic rats was significantly decreased compared with that in the normal control rats. Conversely, diabetic rats treated with *R. nasutus* for 30 days exhibited a noticeable increase in G6PDH activity that was almost equal to that resulting from glibenclamide treatment (Figure 4), indicating that *R. nasutus* has positive effects on diabetes.

## 4. Discussion

Increased LDH activity in diabetic rats has been reported by various researchers [22–24]. In earlier reports, Singh et al. [25] reported that the LDH levels of diabetic rats were higher than those of the control rats and that the elevated LDH levels were associated with decreased insulin secretion. In diabetic animals, the extreme accumulation of pyruvate may lead to higher LDH activity. In the presence of LDH, excessive pyruvate is converted into lactate, leading to increased LDH activity, which could be attributed to the reduced insulin levels in diabetic individuals. Elevated LDH levels were observed in the STZ-induced diabetic rats and are associated with impaired glucose-stimulated insulin secretion [26]. Previous reports also suggest that other herbs such as *Murraya koenigii* and *Ocimum sanctum* can reduce LDH levels in diabetic rats [27]. However, the mechanism is still unclear.

The continuous administration of *R. nasutus* extract for 30 days reduced the LDH activities in diabetic rats in a manner parallel to that induced by glibenclamide. Glibenclamide is an oral antihyperglycemic drug that is used to treat diabetes due to its fast action, relatively low cost, and easy availability. Glibenclamide binds to the surface receptor present on the **β** cells of the pancreas thereby reduces the conductance of the ATP-sensitive potassium channels. The reduction in potassium efflux causes membrane depolarization and the influx of calcium through calcium channels, which eventually causes insulin secretion [28]. The protective role of *R. nasutus *against these effects indicates that this plant is able to prevent the harmful effects of high LDH levels observed in diabetes. 

SDH is one of the most important marker enzymes for mitochondria. Its activity is generally higher than that of other enzymes in both developing and adult animals. As reported by Satav and Katyare [29], the hepatic SDH activity was significantly decreased in STZ-induced diabetic rats. Diminished SDH activity in diabetic rats affects the succinate-fumarate conversion, contributing to depressed oxidative metabolism in mitochondria. It has been suggested that the diabetogenicity of STZ is due to the inhibition of the activities of citric acid cycle enzymes, such as SDH. In the present study, the SDH activity was ameliorated by *R. nasutus* treatment in diabetic rats. The presence of antioxidants, such as phenolic compounds, may have also contributed to this effect, as various antioxidants were confirmed to be present in this plant extract in our study. Further studies to investigate this association and to determine the exact composition of this extract will be useful. It has been reported that the increased SDH activity in diabetic rats treated with plant extracts is indicative of better energy utilization due to the production of intermediates in the tricarboxylic acid cycle [25]. Thus, our findings suggest that there is increased mitochondrial oxidative potential and energy synthesis when diabetic rats are treated with the methanolic extract of *R. nasutus*.

The regulation of ammonia levels in hepatic tissue is impaired in diabetic animals and humans [30]. In this study, the activity of GDH was significantly decreased in the livers of diabetic rats. This decrease in GDH activity may have been due to the instability of energy metabolism, the impairment of glutamate transport, or the activation of lipid peroxidation in the liver. In contrast, diabetic rats treated with *R. nasutus* extract exhibited improvements in GDH activity. The increased levels may be due to the synchronization of energy metabolism and the elevation of glutamate levels in the cells. The ameliorated activities of mitochondrial enzymes and GDH by treatment with a plant extract again suggest that this plant extract has a protective role against diabetes complications because a similar improvement in GDH activity was observed with the continuous administration of a standard antidiabetic drug, glibenclamide, for 30 days. Therefore, *R. nasutus* should be investigated further as a potential antidiabetic herb.

The extramitochondrial enzyme G6PDH is highly specific for NADP as an electron acceptor. The present results showed a dramatic decrease in the levels of G6PDH in the liver tissue of STZ-induced diabetic rats. These results were similar to those of previous studies that also demonstrated lower G6PDH activity in diabetic tissues [31–33]. It has been reported that hyperglycemia decreases the activities of hexose monophosphate shunt enzymes in diabetic animals and decreases G6PDH activity in diabetic rats. The reduced activity of G6PDH affects the NADPH concentrations in cells, thus contributing to oxidative stress, which can lead to diabetic complications [34]. In the present study, the elevated G6PDH activity observed with the continuous administration of *R. nasutus* to diabetic rats may help reduce diabetes-associated complications. The recovery of G6PDH activity in the plant-extract-treated diabetic rats may be due to the antioxidants present in the leaves [35]; these antioxidants include phenolic groups, the presence of which we confirmed through phytochemical analysis. Further studies to identify the exact composition of the extract will be useful in the future.

STZ results in the irreversible destruction of *β* cells and has been widely used to induce type 1 diabetes in experimental animal models. The considerable destruction of *β* cells after STZ injection is purported to be due to the inhibition of free radical scavenging enzymes, thus encouraging the production of various free radicals [36, 37]. This destruction of *β* cells accounts for the marked decrease in the amount of insulin produced by the *β* cells of the pancreas, which in turn affects glucose metabolism. In the present study, the observed significant increase in the blood glucose level in diabetic rats could be due to the destruction of pancreatic *β* cells by STZ, strengthening the hypothesis that STZ induces diabetes via the generation of free radicals. Because the liver performs most of the reactions involved in the synthesis and utilization of glucose, it is plausible that the elevation of the glucose level in STZ-treated rats can be attributed by the oxidative stress produced in the pancreas due to single-strand breaks in the DNA of the pancreatic islets [38]. 

Some of the active constituents of *R. nasutus *have previously been reported. In a study conducted by Sendl et al. [39], rhinacanthin C and D were found to have antiviral activity, with effects comparable to those of ganciclovir and acyclovir. In another study, the *in vitro *antiproliferative activity of rhinacanthin C was reported to be comparable with or slightly weaker than that of the anticancer agent 5-fluorouracil [40]. The researchers also confirmed that the *in vitro *antiproliferative activity of the ethanolic  extract of *R. nasutus *roots was due to rhinacanthin C, whereas the activity of the aqueous extract of the leaves of *R. nasutus *was due to compounds other than rhinacanthin C that are still unknown. Both the aqueous and ethanolicextracts of* R. nasutus *showed *in vivo *antiproliferative activity after daily oral administration once for only 14 days. To our knowledge, no studies have investigated the possible constituents of *R. nasutus* that contribute to the changes in the cytosolic and mitochondrial enzymes levels and can help control diabetes. 

## 5. Conclusion

The continuous administration of *R. nasutus* for 30 days resulted in significant improvements in cytosolic and mitochondrial enzymes levels and activities, and these effects may contribute to the antidiabetic effects of this plant. 

## Figures and Tables

**Table 1:** Phytochemical analysis of various extracts of *R. nasutus*.

Contents	Hexane	Ethyl acetate	Methanol	Water
Steroids	+	+	+	+
Triterpenes	+	+	−	−
Saponins	+	+	−	−
Flavonoids	−	−	+	+
Phenolic compounds	−	−	+	−
Tannins	−	−	+	+
Carbohydrates	−	−	+	+
Glycosides	−	−	−	−

+: present

−: absent.
